# Protocol for the systematic reviews on the desirable and undesirable effects of pharmacological treatments of allergic rhinitis informing the ARIA 2024 guidelines 

**DOI:** 10.5414/ALX02515E

**Published:** 2024-07-22

**Authors:** Rafael José Vieira, Maria Inês Torres, Antonio Bognanni, Sara Gil-Mata, Renato Ferreira-da-Silva, Nuno Lourenço-Silva, António Cardoso-Fernandes, André Ferreira, Henrique Ferreira-Cardoso, João Teles, Miguel Campos-Lopes, João A. Fonseca, Juan José Yepes-Nuñez, Ludger Klimek, Torsten Zuberbier, Holger Schünemann, Jean Bousquet, Bernardo Sousa-Pinto

**Affiliations:** 1 MEDCIDS – Department of Community Medicine, Information and Health Decision Sciences,; 2 CINTESIS@RISE – Health Research Network, MEDCIDS, Faculty of Medicine, University of Porto, Porto, Portugal,; 3 Department of Health Research Methods, Evidence and Impact,; 4 Department of Medicine, Evidence in Allergy Group, McMaster University, Hamilton, ON, Canada,; 5 Unit of Anatomy, Department of Biomedicine, Faculty of Medicine, University of Porto,; 6 Department of Ophthalmology, Centro Hospitalar Universitário do Porto, Porto, Portugal,; 7 School of Medicine, Universidad de los Andes, Bogotá, Colombia,; 8 Department of Otolaryngology, Head and Neck Surgery, Universitätsmedizin Mainz, Mainz,; 9 Center for Rhinology and Allergology, Wiesbaden,; 10 Institute of Allergology, Charité – Universitätsmedizin Berlin, Corporate Member of Freie Universität Berlin and Humboldt-Universität zu Berlin,; 11 Fraunhofer Institute for Translational Medicine and Pharmacology ITMP, Allergology and Immunology, Berlin, Germany, and; 12 ARIA, Montpellier, France

**Keywords:** allergic rhinitis, allergy, antihistamines, corticosteroids, quality of life

## Abstract

There is insufficient evidence regarding the comparative efficacy and safety of pharmacological treatments of allergic rhinitis (AR). In the context of informing the 2024 revision of the Allergic Rhinitis and its Impact on Asthma (ARIA) guidelines, we plan to perform three systematic reviews of randomized controlled trials (RCTs) comparing the desirable and undesirable effects (i) between intranasal and oral medications for AR; (ii) between combinations of intranasal and oral medications versus nasal or oral medications alone; and (iii) among different intranasal specific medications. We will search four electronic bibliographic databases and three clinical trials databases for RCTs examining patients ≥ 12 years old with seasonal or perennial AR. Assessed outcomes will include the Total Nasal Symptom Score, the Total Ocular Symptom Score, and the Rhinoconjunctivitis Quality-of-Life Questionnaire. We will assess the methodological quality of included primary studies by using the Cochrane risk-of-bias tool. If appropriate, we will perform a pairwise random-effects meta-analysis for each pair of assessed medication classes and outcomes, as well as a network meta-analysis to assess the comparative efficacy of intranasal medications among each other. Heterogeneity will be explored by sensitivity and subgroup analyses. This set of systematic reviews will allow for a comprehensive assessment of the effectiveness and safety of pharmacological interventions for AR and inform recommendations in the context of the ARIA guidelines.

## Introduction 

Allergic rhinitis (AR) is a highly prevalent disease that poses a significant burden for society as it may lead to reduced quality of life as well as impaired academic and work productivity [1, 2, 3, 4]. The current mainstay of pharmacological interventions for AR includes intranasal corticosteroids, intranasal antihistamines, fixed combinations of corticosteroids and antihistamines, oral antihistamines, or leukotriene receptor antagonists. The 2016 revision of the Allergic Rhinitis and its Impact on Asthma (ARIA) guidelines addressed the pharmacological treatment of AR [5]. However, for many cases, the level of evidence was reported to be “low” or “very low” [5]. This was partly due to insufficient evidence regarding several aspects of the pharmacological treatment of AR, such as whether effectiveness differences may exist (i) between intranasal and oral medications, (ii) between combined intranasal and oral medications versus oral medications alone, and (iii) among different intranasal specific medications. Indeed, while there have been previous systematic reviews attempting to answer these questions, they have important limitations. Three recent systematic reviews assessed the combination of intranasal corticosteroids and oral antihistamines versus intranasal corticosteroids alone, but the effectiveness of these combinations versus oral antihistamines alone was not assessed [6, 7, 8]. In addition, two of these systematic reviews restricted the search to two electronic databases and failed to search gray literature [6, 8]; while the other (i) did not include studies published in journals not indexed in the Scientific Citation Index, (ii) did not incorporate a comprehensive range of variations, and (iii) failed to use validated search filters in their search strategy, potentially impacting the comprehensiveness of the surveyed literature [7]. One systematic review assessed the comparison between intranasal and oral medications but limited their search to articles published after 2003 and excluded studies based on language [9]. Finally, to the best of our knowledge, there are no systematic reviews assessing the direct comparison of different formulations of intranasal medications. 

In the context of developing the 2024 revision of the ARIA guidelines (focusing on the pharmacological treatment and management of AR), and considering the aforementioned literature gaps, we identified the need for conducting three systematic reviews to adequately assess the desirable and undesirable effects of the pharmacological treatments of AR. Specifically, we will perform systematic reviews with meta-analyses comparing the effectiveness (i) between intranasal and oral medications for AR; (ii) between combined intranasal and oral medications versus nasal or oral medications alone; and (iii) among different intranasal specific medications. These systematic reviews will complement a fourth one conducted by our team and whose protocol has already been published [10]. 

## Materials and methods 

We will perform three systematic reviews of randomized controlled trials (RCTs) in patients with AR, comparing the effectiveness of:

- Systematic review #1: AR intranasal versus oral medications (PROSPERO registration number: CRD42023495296);
- Systematic review #2: combination of AR intranasal and oral medications versus nasal or oral medications alone (PROSPERO registration number: CRD42023495354);
- Systematic review #3: intranasal corticosteroids, intranasal antihistamines, and intranasal fixed combinations of corticosteroids + antihistamines (PROSPERO registration number: CRD42023495363).

Combination between oral antihistamines and oral or nasal decongestants will not be studied in these systematic reviews, due to emerging recommendations against their use [11]. 

These systematic reviews will follow the Preferred Reporting Items for Systematic Reviews and Meta-Analyses (PRISMA) guidelines [12] for systematic reviews #1 and #2, and the PRISMA extension for network meta-analyses (PRISMA-NMA) [13] for systematic review #3. 

### Eligibility criteria 

For all of the systematic reviews, we will include RCTs with a parallel design assessing patients ≥ 12 years old with seasonal or perennial AR with results on at least one of the following patient-reported outcome measures: Total Nasal Symptom Score (TNSS), Total Ocular Symptom Score (TOSS), Total Symptom Score (TSS), or Rhinoconjunctivitis Quality of Life Questionnaire (RQLQ). We define the TNSS as any score computed based on the sum of several patient-reported scores for individual nasal symptoms, and the TOSS as any score computed based on the sum of several patient-reported scores for individual ocular symptoms. Accordingly, we define the TSS as the combination of different types of rhinitis symptoms (e.g., nasal, ocular and/or other symptoms). This may be especially important as studies may use different definitions for the TNSS, TOSS, or TSS (e.g., many antihistamine trials consider the TNSS as the sum of three nasal symptoms and do not include congestion, as opposed to other trials). We will consider the TNSS, TOSS, and TSS assessed in a reflective manner; that is, reflecting patients’ symptoms in the previous 12 or 24 hours. Considering Food and Drug Administration recommendations [14], we will only include RCTs with a follow-up period of at least 2 weeks if assessing patients with seasonal AR or at least 4 weeks if assessing patients with perennial AR. 

We will categorize medications as follows, clustering them by means of administration:

- Intranasal medications: We will consider intranasal corticosteroids (beclomethasone, budesonide, ciclesonide, fluticasone furoate, fluticasone propionate, mometasone furoate, and triamcinolone), intranasal antihistamines (azelastine, levocabastine, and olopatadine), and fixed combinations of intranasal corticosteroids + antihistamines (azelastine–fluticasone and olopatadine–mometasone).
- Oral medications: We will consider oral antihistamines (astemizole, azelastine, bilastine, cetirizine, chlorpheniramine, cyproheptadine, clemastine, desloratadine, dexchlorpheniramine, dimetindene, diphenhydramine, ebastine, epinastine, fexofenadine, hydroxyzine, levocabastine, levocetirizine, loratadine, promethazine, and terfenadine) and oral leukotriene receptor antagonists (montelukast and zafirlukast).

The interventions and comparators to be considered will differ depending on the systematic review: for systematic review #1, we will consider primary studies assessing both intranasal and oral medications; for systematic review #2, we will include primary studies assessing both the combination of intranasal and oral medications and oral medications or nasal medications alone; for systematic review #3, we will consider solely studies assessing intranasal medications (performing direct comparisons of intranasal medications or intranasal medications versus placebo). 

We will not exclude studies based on publication language, date, or status (i.e., we will include relevant studies irrespective of whether they were published as full papers, conference abstracts, clinical trials registries, or others). 

### Information sources and search strategy 

We will search studies for the three systematic reviews using a search strategy designed for a previous systematic review [10], conducted by our team. We will search bibliographic databases (MEDLINE and Embase via Ovid, Web of Science, and the Cochrane Central Register of Controlled Trials) in addition to manually searching the clinicaltrials.gov, the GSK clinical study dataset, and the AstraZeneca Clinical Trials Website in order to identify potentially unpublished trials. Content experts within the ARIA guidelines development group will be asked to check the final list of included studies for completeness. 

### Study selection and data collection 

Each record will be independently reviewed in pairs, first by title and abstract screening, then by assessing the full-text for eligibility. Any non-excluded record will be assessed to determine whether multiple publications originated from the same study. Data from each included primary study will be independently extracted by two reviewers using a dedicated online form. From each study, in addition to identifying information, we will retrieve data on (i) the assessed disease (seasonal or perennial AR; for studies assessing both patients with seasonal and perennial AR, we will retrieve data separately for these conditions), (ii) the participants’ eligibility criteria, (iii) the data collection period, (iv) the places from where patients were recruited, (v) the follow-up period, (vi) the assessed medications, (vii) the total daily dose of medications, (viii) the number of randomized participants, (ix) the number of participants completing the study, (x) the participants’ age and sex distribution, and (xi) the assessed outcomes. For each desirable effects outcome (TNSS, TOSS, TSS, and/or RQLQ), we will retrieve (i) information on the scale and computation methods, as well as (ii) baseline values and post-intervention and/or change from baseline values. Information on undesirable outcomes will also be retrieved, namely on the frequency of patients (i) developing at least 1 adverse event (AE) (as defined by the authors), (ii) developing at least 1 serious AE, and (iii) discontinuing treatment due to AE. Whenever results are only provided in graphical form (rather than numerical data in text form), estimates will be obtained using the PlotDigitizer tool. Disagreements between reviewers in data selection or extraction will be resolved by consensus or by a third reviewer. We will contact the authors of included primary studies whenever further information is required. Data from multiple reports of the same study will be combined to ensure comprehensiveness, while, if a report provides information on multiple studies, the latter will be considered independently. 

### Risk of bias and critical appraisal of the evidence 

The risk of bias of each included primary study will be independently assessed at an outcome level by two reviewers using the Cochrane risk-of-bias tool [15]. Disagreements will be resolved by consensus or by a third reviewer. 

Certainty in the body of evidence for the different outcomes will be assessed using the GRADE approach [16] or, for the network meta-analysis, the modified GRADE approach for network meta-analysis. In both cases, for the assessment of inconsistency and imprecision, we will follow a minimally contextualized approach [17], using the following minimal important differences (MIDs): for RQLQ, we will consider a MID of 0.5 [18]; and for the TNSS on a 0 – 12 scale, we will consider a MID of 0.28 [19]. 

### Synthesis of the evidence 

All desirable effects outcomes to be assessed are continuous. Therefore, we will present the mean (± standard deviation) baseline and change-from-baseline values for such outcomes. When information on spread measures is missing, this will be estimated based on the approach proposed by Weir et al. [20] as previously described in another systematic review conducted by our team [10, 20]. Undesirable effects outcomes to be assessed are dichotomous, therefore, we will present them by providing information on the absolute frequency of events and on their incidence rate per person-weeks. 

**Pairwise meta-analysis**

We will perform pairwise meta-analysis comparing intranasal versus oral medications, combination of intranasal and oral medications versus oral or nasal medications alone, and direct comparisons of intranasal medications. For desirable effects outcomes presented in the same scale across studies, we will perform random-effects meta-analyses of mean differences (MDs) in change-from-baseline values, while for outcomes presented in different scales, we will perform random-effects meta-analyses of standardized mean differences (SMDs). For outcomes calculated based on the same symptoms but with results presented in different scales, scale reconversion will be performed. For undesirable effects outcomes, we will perform random-effects meta-analyses of incidence rate ratios (IRRs). 

For systematic reviews #1 and #2, meta-analyses will be performed grouping drugs by means of administration (i.e., comparing all intranasal medications versus all oral medications) and by drug class; for systematic review #3, meta-analysis will be performed by individual drug. Separate meta-analyses will be performed for patients with seasonal and perennial AR. 

The restricted maximum likelihood approach will be used to estimate between-study variance. Heterogeneity will be assessed by estimating the p-value of the Q-Cochran test and by the I^2^ statistic. A p-value < 0.10 and an I^2 ^≥ 50% will be considered to represent substantial heterogeneity. Irrespective of the findings, we will further explore heterogeneity to identify potential sources of variations. We will conduct sensitivity analyses to assess the impact by risk of bias on the pooled effect estimates. Subgroup analyses will also be performed in relation to the follow-up period of the study and to the dose of the drug being assessed. The a priori hypotheses underlying these subgroup analyses is that a higher effect may be observed when higher doses of the drug are tested but not necessarily when assessing longer follow-up periods. For subgroup analyses suggesting the existence of effect modification, the credibility of such effect modification will be assessed using the Instrument to assess the Credibility of Effect Modification Analyses (ICEMAN) in a meta-analysis of RCT tools [21]. 

We will apply a complementary approach using Bayesian methods to compute the probability of each intervention being more effective tha**n** the comparison in improving efficacy outcomes more than the MID and/or of established decision thresholds. For each comparison, we will perform Bayesian random-effects meta-analyses of mean differences in change-from-baseline values of the TNSS and RQLQ using non-informative prior distributions (dnorm(0, 0.00001) for the effect size measures and dunif(0,100) for the tau parameter). We will run a minimum of 200,000 iterations with a burn-in of 100,000 sample iterations until convergence is reached (as assessed by an effective sample simple size of at least 4,000 and a Gelman-Rubin statistic < 1.01). We will obtain the posterior predictive probability distributions for the effect size measures of each primary study and of the pooled meta-analytical values. For each medication and outcome, we will quantify the proportion of simulations of effect size measures (i) that are higher than the MID (this analysis will not be performed for the TOSS on account of a lack of an established MID), and/or (ii) that fall within each interval defined by established decision thresholds (i.e., displaying a trivial, small, moderate, or large effect). The latter approach will also be applied in relation to safety outcomes. 

**Network meta-analysis**

To assess the comparative efficacy of intranasal medications, including direct and indirect comparisons, we will perform a network meta-analysis for each outcome in systematic review #3. 

For desirable effects outcomes, we will perform random-effects network meta-analysis of MD or SMD in change-from-baseline values, depending on whether or not outcomes are presented in the same scale across studies. For undesirable effects outcomes, we will perform random-effects network meta-analyses of IRR. Network meta-analyses will be performed by individual drug and by drug class. 

The transitivity assumption (i.e., the existence of comparable distributions of patient characteristics across studies in the treatment network) will be assessed by considering patient and study characteristics across the studies that compare pairs of treatments. The coherence assumption (i.e., the agreement in estimates between direct and indirect sources of evidence) will be assessed both by local and global approaches. First, we will assess local coherence using the Separating Indirect from Direct Evidence (SIDE) approach as proposed by Dias et al. [22]. This method evaluates the incoherence factor for every pairwise comparison in a network by contrasting a direct estimate (when available) with an indirect estimate; the latter being estimated from the entire network once the direct evidence has been removed [23]. Finally, we will assess heterogeneity by estimating the p-value of the Q-Cochran test and by the I^2^ statistic. A p-value < 0.10 and an I^2^ ≥ 50% will be considered to represent substantial heterogeneity. 

All analyses will be performed using software R, with the use of the metafor, meta, and netmeta packages. 

The synthesized evidence, together with the confidence in evidence ratings and considerations for each domain, will be summarized in dedicated Evidence Profiles using GRADEpro software (www.GRADEpro.org) [24]. 

## Discussion 

There are some limitations to these systematic reviews. First, we will consider only patients aged 12 years or older, therefore our future conclusions may not be generalizable to children. However, this may be overcome by a future systematic review in pediatric patients. Additionally, we will rely on the search strategy for a previous systematic review on intranasal antihistamines and corticosteroids. However, the three systematic reviews will include studies solely if they assess intranasal medications (whether alone or in combination with oral medications). From an efficacy point of view, we will include studies only if they assess the TNSS, TOSS, TSS, or RQLQ. This methodological choice stems from the fact that the main symptoms associated with AR are the nasal or ocular ones. Therefore, we will focus on these symptoms and on their impact on quality of life. Another limitation is that, although non-randomized studies may offer complementary information on the effectiveness of interventions [17], we will not include non-randomized studies in our systematic review. 

There are also limitations related to the available evidence. Heterogeneity may exist in outcome definition across studies. For example, for the TNSS, while some studies may calculate it by considering the sum of three nasal symptoms, others base it on the sum of four. To address this variation in outcome definitions during meta-analysis, we plan to conduct separate meta-analyses for differently defined outcomes. Finally, for the first two systematic reviews (i.e., comparing AR intranasal versus oral treatments, or the combination of AR intranasal and oral medications versus nasal or oral medications alone), the number of published studies per specific drug may be too small, prompting the need for meta-analyses to be done grouping drugs by class and means of administration. 

This set of systematic reviews has been devised to inform the 2024 revision of the ARIA guidelines on the desirable and undesirable effects of the pharmacological treatments of AR. These are two of the criteria in the GRADE Evidence to Decision framework, which will be adopted in the context of the aforementioned guidelines [25]. Performing adequate judgements on these criteria (as well as on the balance between desirable and undesirable effects) is key in the process of formulating guideline recommendations, even though additional criteria (such as patients’ values and preferences or resource use) also need to be taken into account. 

Beyond the ARIA guidelines, this set of systematic reviews will be particularly relevant, as they will enable (i) a comprehensive assessment of the effectiveness of intranasal medications (whether alone or in combination with oral medications) versus oral medications, as well as (ii) a direct and indirect comparision of the effectiveness of different intranasal medications for AR treatment. Furthermore, it will enable us to assess the methodological quality of published studies and the reliability of the body of existing evidence, allowing us to identify aspects that future primary studies should consider. The comprehensiveness in scope and the adopted methodology will allow these systematic reviews to fill in relevant literature gaps and overcome some of the limitations of previous systematic reviews. 

## Authors’ contributions 

RJV, MIT, BSP, AB, SGM and JB participated in the study design and in the drafting of the protocol manuscript. RFS, NLS, ACF, AF, HFC, JT, MCL, JAF, JJYN, LK, TZ and HS participated in the study design and in the critical revision of the manuscript. 

## Funding 

The work of this study has been funded within the context of the Allergic Rhinitis and its Impact on Asthma (ARIA) group. 

## Conflict of interest 

LK reports grants from Allergopharma, MEDA/Mylan, ALK Abelló, LETI Pharma, Stallergenes, Sanofi, ASIT biotech, Lofarma Quintiles, AstraZeneca, GSK and Inmunotk, and personal fees from Allergopharma, MEDA/Mylan, HAL Allergie, LETI Pharma, Sanofi, Allergy Therapeut and Cassella Med, outside the submitted work. JAF reports grants from Astrazeneca and Mundipharma; and personal fees from AstraZeneca, Mundipharma, Sanofi, GSK and Teva, outside the submitted work. TZ reports grants from Novartis and Henkel; personal fees from Bayer Health Care, FAES, Novartis, Henkel, AstraZeneca, AbbVie, ALK, Almirall, Astellas, Bencard, Berlin Chemie, HAL, Leti, Meda, Menarini, Merck, MSD, Pfizer, Sanofi, Stallergenes, Takeda, Teva, UCB, Kryolan and L’Oréal, outside the submitted work. JB reports personal fees from Chiesi, Cipla, Hikma, Menarini, Mundipharma, Mylan, Novartis, Purina, Sanofi-Aventis, Takeda, Teva and Noucor and other from Kyomed-Innov, outside the submitted work. 
